# Short‐ and Long‐Term Reciprocal Bone‐Muscle Relationships in the Upper and Lower Extremity of Afro‐Caribbean Men by DXA: A Longitudinal Analysis of the Tobago Bone Health Study

**DOI:** 10.1002/jbm4.10406

**Published:** 2020-10-01

**Authors:** Andy KO Wong, Anthony Pokhoy, Abinaa Chandrakumar, Ryan K Cvejkus, Joseph M Zmuda

**Affiliations:** ^1^ Centre of Excellence in Skeletal Health Assessment, Joint Department of Medical Imaging University Health Network Toronto Ontario Canada; ^2^ Toronto General Hospital Research Institute, University Health Network Toronto Ontario Canada; ^3^ Division of Epidemiology, Dalla Lana School of Public Health University of Toronto Toronto Ontario Canada; ^4^ Department of Epidemiology Graduate School of Public Health, University of Pittsburgh Pittsburgh PA USA

**Keywords:** AFRO‐CARIBBEAN, BONE–MUSCLE INTERACTIONS, DXA, LEAN MASS, MEN

## Abstract

Little is known about the time course of muscle–bone effects and whether a reciprocal clinical effect of bone on muscle is present. We hypothesized that lean mass (LM) measures at the arms and legs have a stronger relationship with BMD measured within the same region than the reciprocal effect. The Tobago Bone Health Study was used to address this hypothesis, examining body composition data from total body DXA scans obtained at 0, 48‐, and 120‐month visits. A longitudinal analysis of LM, LM/height^2^ (LMI), and LM/BMI was conducted at the upper and lower extremities separately, in relation to BMD within the corresponding region. A cross‐lagged panel model was used to study pathways from 0 to 120 months for muscle–bone and bone–muscle effects within the same visit, and across each lagged period. Models accounted for age, height, weight, race, arthritis, prior nontraumatic fracture after age 40, number of units of alcohol consumed per week, current smoking, diagnosis of diabetes mellitus, amount of walking in the last week, grip strength, and hospitalizations. Significant models demonstrating parsimony, and meeting absolute and relative fit criteria were retained. Among 1286 Afro‐Caribbean men (mean age: 53 ± 9 years, BMI: 27.43 ± 4.23 kg/m^2^) with data available for all visits, LM, LMI, and LM/BMI had modest contemporaneous relationships with BMD, which dissipated with lagged time. The size of these effects was stronger at the legs than at the arms. These lagged effects were primarily mediated through indirect same time‐point muscle–bone relations rather than a true directly lagged effect. Bone density showed only a small effect on LM arm measures across lagged time, but this was impossible to tease‐out from same time‐point relations. These results suggest muscle–bone relationships are not long‐lasting at least beyond 48 months. Efforts to maintain muscle and bone strength should focus on shorter‐term interventions. More studies are needed with serial bone–muscle imaging over shorter periods. © 2020 The Authors. *JBMR Plus* published by Wiley Periodicals LLC on behalf of American Society for Bone and Mineral Research.

## Introduction

Osteoporosis has traditionally been assessed by measurements of BMD, focusing clinical care around *T*‐scores measured at the hip and spine. However, bone is not an isolated structure; it is highly influenced by external factors such as hormones, fat, and muscle. Osteosarcopenia is a relatively new characterization that recognizes patients who are experiencing a concurrent diagnosis of osteoporosis and sarcopenia^(^ ^1^ ^)^; this multidimensional understanding of bone and overall musculoskeletal health is beginning to influence clinical practice. In fact, groups have already proposed guidelines for the diagnosis of sarcopenia.^(^ ^2^, ^3^, ^4^ ^)^ A further understanding of the bone–muscle relationship in normal and diseased profiles may provide a more complete picture of musculoskeletal health.

Following Wolff's law and mechanostat theory, mechanical loading on bone influences its structure and strength over time.^(^ ^5^ ^)^ Muscle is one of the largest of these voluntary mechanical loads acting on bones. By virtue of its attachment to bone surfaces through tendons, muscle forces stimulate the mechanosensing machinery within osteocytes to drive bone remodeling.^(^ ^6^ ^)^ Various descriptions of muscle characteristics and their potential impact on bone properties have been published. Ritwegger and colleagues showed significant correlations (*N* = 92, *p* < 0.001) in the cross‐sectional area of bone and muscle at various levels of the lower tibia (4%, 14%, 33%, and 66% from the distal tibia) using pQCT.^(^ ^7^ ^)^ A 5‐year longitudinal analysis of Koreans aged ≥65 years (*N* = 337) showed an association between a greater decline of total hip BMD and initial lower leg lean mass (*r* = 0.205, *p* < 0.05).^(^ ^8^ ^)^ The effects of muscle on bone also have a molecular basis as a number of muscle‐secreted cytokines (myokines) have been identified in bone remodeling.^(^ ^9^, ^10^, ^11^ ^)^ This association is not unidirectional, however, as bone may release factors that are involved in myogenesis and influence muscle function. For example, Huang and colleagues have shown that C2C12 muscle cells, conditioned in MLO‐Y4 osteocyte‐like cell conditioned media, increased myogenic differentiation and ex vivo soleus muscle contractile force.^(^ ^12^ ^)^ Prostaglandin E2, another factor secreted by osteocytes, has been shown to induce myogenesis of C2C12 myoblasts.^(^ ^13^ ^)^ There has been a lack of in vivo evidence on the potential effect that bone may have on muscle.

Clinically, muscle and bone have been measured using CT, MRI, and DXA.^(^ ^14^ ^)^ Although CT and MRI yield more detailed information regarding bones and muscles,^(^ ^15^, ^16^ ^)^ respectively, neither is currently used routinely as part of the standard of care for managing patients with osteoporosis, or to evaluate older patients for sarcopenia and physical frailty. More crude estimates of bone and muscle are possible with DXA. However, its ability to rapidly image the full body is a major advantage, lending to the capacity to explore bone–muscle relationships within specific regions of the musculoskeletal anatomy. Appendicular lean mass (ALM), the ALM index (ALM/height^2^ [ALMI]), and ALM/ BMI have been used to represent the amount of soft tissue within the arms and legs, with and without division over an estimate of the body's surface area or level of obesity (BMI), respectively.^(^ ^17^ ^)^

To date, there have been a number of cross‐sectional studies and some longitudinal studies that identify muscle's effect on bone, as well as studies that show the biochemical basis for this interaction. Bone's effect on muscle is a recent area of research and is less well‐defined, existing as mostly in vitro studies of bone and muscle cell lines. Moreover, there is a lack of studies that concurrently show the reciprocal bone–muscle effects in a longitudinal setting. An additional gap in the literature is the few studies focused on men. Sheu and colleagues have already shown that a history of diabetes mellitus and prostate cancer are prominent risk factors for trabecular and cortical bone density in men of African descent.^(^ ^18^ ^)^ They have also determined that muscle–fat infiltration is prominent among the same group of men.^(^ ^19^ ^)^ However, it is unclear how muscle further contributes to long‐term bone maintenance, and whether reciprocal effects are important for mutual preservation. Therefore, the present study aimed to explore short‐ and long‐term reciprocal bone–muscle relationships using cross‐lagged panel modeling of DXA imaging outcomes in a cohort of Afro‐Caribbean men from the Tobago Bone Health Study. We hypothesized that baseline LM, LMI, and LM/BMI from DXA have a stronger effect on BMD measured within the same region than the effect that BMD has on LM, LMI, and LM/BMI, as measured over time. We further postulated that long‐term effects of LM, LMI, and LM/BMI on bone are weaker than short‐term effects.

## Participants and Methods

### Study design

The present study is a longitudinal cross‐lagged analysis of data collected from three waves of the Tobago Health Study, whose original objective was to investigate prostate cancer screening. Details of this cohort have been previously described.^(^ ^19^, ^20^ ^)^

### Study recruitment and data selection

Study baseline (V0) was between 1997 and 2003, recruiting 3170 ambulatory, community‐dwelling men 40 years of age or older within the dual‐island Caribbean nation Trinidad and Tobago by convenience sampling through various media. The recruited sample represented approximately 60% of all age‐eligible men on the islands.^(^ ^21^ ^)^ In 2004, these men were invited to participate in a bone‐specific extension study to understand risk factors for accelerated bone loss over 6 years using DXA, and an additional 451 new participants were recruited. The present study used V0, V3, and V5 data, corresponding to approximately 0, 48‐, and 120‐month postbaseline follow‐up, respectively. A total of 2693, 2493 (representing 69.1% of survivors), and 1791 (representing 77.6% of survivors) participants completed V0, V3, and V5 DXA scans, respectively, where 1340 participants had data for all three visits.

### DXA body composition

DXA scans were acquired at 70‐ to 140‐kV pulsed synchronously per line of pixels precalibrated to density values on a Hologic QDR 4500 W densitometer (Hologic, Bedford, MA, USA) by a trained and certified technologist. Weekly quality control was performed using a whole‐body phantom air scan to ascertain scan quality from machine drift, as monitored by Synarc (Waltham, MA, USA). For whole‐body scans, ALM and total‐body LM were obtained as surrogates of muscle mass, as has been examined in a number of large epidemiological studies^(^ ^22^, ^23^, ^24^, ^25^ ^)^ with validation against MRI (*R* ^2^ = 0.96, *p* < 0.01)^(^ ^26^ ^)^ and true ex vivo mass from porcine tissues.^(^ ^27^ ^)^ By default, the left side was used in analyses where paired anatomical sites were available, unless there was a prosthesis or fracture at the site of interest, in which case the contralateral side was used. Total‐body measures were computed without head measurement. For each region, LM, fat mass (FM), BMD, and BMC were computed. Appendicular LM^(^ ^22^, ^23^ ^)^ and BMD were calculated separately for upper and lower limbs. The analyses performed here included results for the upper and lower limbs, and both appendicular sites.

### Anthropometrics, medical history, and medication use

Information about age and ethnicity were retained from baseline. Height and weight were obtained at each visit using a standiometer and standard scale, respectively. Use of glucocorticoids and lifestyle questions about current smoking and units of alcohol per week within the last 12 months were collected. Participants were asked if they walked for exercise, going to destinations outside of the home, or walking the dog at least 10 times in the past 12 months, whether they did go walking within the last 7 days, the frequency of walking over these 7 days, and how much time was spent walking during this period. This information was combined to yield an estimate of the amount of walking per week. Grip strength was measured twice in both hands using a Jamar dynamometer, and averaged across all measurements. Self‐report of fractures after age 40, diagnosis of diabetes mellitus, and arthritis were determined at V0. History of hospitalization (yes/no) was obtained at V3 and V5 for cases related to heart attack, stroke, transient ischemic attacks, congestive heart failure, intermittent claudication, or angina. All the noted variables were considered for covariate analysis in models.

### Statistical analysis

The effects of muscle on bone and bone on muscle across the three waves of data were evaluated using cross‐lagged panel models (CLPMs). Our a priori hypothesis focused on LMI primarily of the lower extremity and its effect on BMD within the same region, with larger effects expected within the same time point than at cross‐lagged periods. Model specification and identification was tested to ensure adequate degrees of freedom (df). To enable successful convergence, the relationships among V0, V3, and V5 muscle; and the corresponding relationships for bone, were each constrained to be equal, thus boosting df for improved statistical power. We tested the validity of these constraints in univariate GLMs and in unconstrained and unadjusted models. Within‐visit bone–muscle relationships were also modeled and compared with cross‐lagged effects. All CLPMs accounted for age, height, weight, current smoking, units of alcohol per week within the last 12 months, amount of walking per week, grip strength, history of hospitalization, self‐report of fractures after age 40 years, diagnosis of diabetes mellitus, and arthritis. Because only one individual showed use of glucocorticoid, he was excluded and glucocorticoid use was not entered into models. Model fit was evaluated using absolute, parsimonious, and relative fit indices including chi‐square (χ^2^), standardized root mean residual (SRMR), root mean square error of approximation (RMSEA), and Bentler–Bonett normed fit index (BB‐NFI). Adequate fit was defined here as having any two of a nonsignificant χ^2^ test, RMSEA upper CI under 0.08, an SRMR under 0.05, or BB‐NFI over 0.95 as suggested by Hu and Bentler.^(^ ^28^ ^)^ All models were conducted for the leg, arm, and all appendicular sites with or without adjustment for height squared or BMI (LM, LMI, or LM/BMI). Because of the potential mediation pathways generated, a formal mediated indirect versus direct effect was measured. Where convergence failed or where analyzed matrices were not positive definite, the quasi‐Newtonian method was used and number of function calls were increased. A complete case analysis was conducted without the need for handling missing data.

In post hoc analyses the effect of baseline LM or BMD measures on subsequent changes in BMD or LM, respectively, over V3 to V5, adjusting for baseline BMD or LM was evaluated using a general linear model, reporting regression coefficients and 95% CIs. The same covariates described above were included in the model. These sensitivity analyses served multiple purposes: (1) to evaluate muscle–bone effects on future changes, which the CLPMs were unable to do; (2) to separately test bone or muscle effects on within‐metric muscle or bone changes; and (3) to enable comparison with previous studies that had performed similar evaluations.

Power was considered for these analyses based on a previous simulation of CLPMs by Wu and colleagues^(^ ^29^ ^)^ demonstrating that a sample of 500 was sufficient to study mediation across three cross‐lagged waves of data, a power of 0.80, type I error robust to 0.025 to 0.075, and a ratio of exposure mediator to mediator‐outcome effects of 0.30, yielding Akaike's information criterion of over 0.80. Based on a sample size above 1200, it is anticipated that our analyses were adequately powered.

## Results

In this Tobago cohort of 1341 men who completed all follow‐up visits, 1286 had complete data including covariates. Mean age was in the middle‐age range and BMI within the overweight category (Table 1). The majority reported African ancestry with only few individuals having experienced a prior fracture. At baseline, 65 (4.8%) participants had osteoporosis, which increased to 390 (29.1%) by V3, and 601 (44.8%) had osteoporosis by V5. Only one individual reported use of glucocorticoids, but he was excluded from analyses because of potential outlier effects. The median follow‐up time from baseline to V3 was 53 (interquartile range [IQR], 47–57) months and from V3 to V5 was 74 (IQR, 67–77) months, yielding a total follow‐up time of 127 (IQR, 117–134) months. Mean arm and leg composition measures with and without adjustment for height squared or BMI showed general declines over time that were significant compared with baseline, with adjustment for repeated measures (Table 2). Measures that combined all information from both arms and legs yielded the smallest declines over time. For each anatomical site, the largest declines occurred between V3 and V5, suggesting the potential for accelerated bone–muscle loss over a decade. The average mean percentage change over the 10 years of the study amounted to: leg LM = −7.6%, leg LMI = −7.7%, leg LM/BMI = −7.5%, leg BMD = −0.90%, arm LM = −7.6%, arm LMI = −7.8%, arm LM/BMI = −8.1%, and arm BMD = −1.9%.

**Table 1:** Participant Characteristics for 1286 Afro‐Caribbean Men With Complete Data

	V0		V3		V5	
Variable	Mean	SD	Mean	SD	Mean	SD
Age (years)	53	9	57	9	63	9
Height (cm)	175.4	6.8	175.5	6.9	175.6	6.9
Weight (kg)	84.52	14.32	85.09	15.11	84.67	14.80
BMI (kg/m^2^)	27.43	4.23	27.55	4.42	27.43	4.32
Total hip BMD *T*‐score	−0.3	0.9	−0.3	0.9	−0.3	1.0
Femoral neck BMD *T*‐score	−0.5	0.9	−0.6	0.9	−0.7	0.9

Variable (overall)	Frequency	%
Fracture after age 40	6	0.47
Alcohol ≥4 units/week	134	10.43
Current smoker	126	9.80
Diabetes mellitus (type I or II)	113	8.83
Arthritis	123	9.64

V0 = baseline visit; V3 = 48‐month visit; V5 = 120‐month visit.

**Table 2:** Comparison of Bone and Muscle Properties Over Time

	V0		V3		V5	
Variable	V0 mean	SD	V3 mean	SD	V5 mean	SD
Leg LM (kg)	10.58	1.54	10.42	1.55	9.77	1.51
Leg LMI (kg/m^2^)	3.43	0.42	3.38	0.43	3.17	0.42
Leg LM/BMI (kg/ kg/m^2^)	0.389	0.050	0.382	0.500	0.360	0.048
Leg BMD (g/cm^2^)	1.403	0.146	1.381	0.146	1.390	0.166
Arm LM (kg)	4.38	0.77	4.33	0.80	4.05	0.78
Arm LMI (kg/m^2^)	1.42	0.23	1.40	0.24	1.31	0.23
Arm LM/BMI (kg/ kg/m^2^)	0.161	0.023	0.158	0.024	0.148	0.023
Arm BMD (g/cm^2^)	0.920	0.084	0.912	0.083	0.903	0.090
Total App BMD (g/cm^2^)	1.209	0.110	1.199	0.113	1.195	0.122
ALM (kg)	29.92	4.31	29.50	4.40	27.87	4.40
ALMI (kg/m^2^)	9.71	1.20	9.57	1.23	9.03	1.21
ALM/BMI (kg/ kg/m^2^)	1.100	0.134	1.080	0.135	1.025	0.134

Comparisons for each measure between time points were made with adjustment for repeated measurement. Arm and leg composition measures at all time point measures were significantly different from one another (*p* < 0.001 for all comparisons). ALM = appendicular lean mass; ALMI = appendicular lean mass index; App = appendicular; LM = lean mass; LMI = lean mass index; V0 = baseline visit; V3 = 48‐month visit; V5 = 120‐month visit.

All CLPMs that adjusted for the full set of baseline covariates and constrained same–variable repeated measure correlations were properly identified (df = 5) and successfully converged with no errors. Although the χ^2^ test was only nonsignificant (indicative of adequate fit) for arm‐related LM and LMI models, LM/BMI models at all sites showed adequate fit based on χ^2^ comparison with the saturated model. All other fit statistics (absolute, relative, and parsimonious fit) were significant for all models (Table 3). Path coefficients between DXA‐derived lean mass measures and bone density measures were, in general, modest (<0.10 difference in BMD per 1 kg difference in LM), even for within time‐point relations (Tables 4, 5, 6). However, same time‐point muscle–bone coefficients were as much as threefold to sixfold larger than cross‐lagged estimates over 53 months or over 74 months. Constrained correlations between repeated bone measures and repeated muscle measures over the three waves were close to 1.0. These constraints were validated for LM, LMI, and LM/BMI at the arms and legs (*r* > 0.90 overall, *r* > 0.80 for arm V3➔V5 and all LM/BMI measures,), BMD at the arms and legs (*r* > 0.90), and ALM, ALMI, and ALM/BMI (*r* > 0.90 overall, except *r* > 0.80 for ALM/BMI) using univariable linear regression models (in all cases, *p* < 0.001).

**Table 3:** Fit Statistics for Fully Adjusted Models Examining Lean Mass Measures

Correlates at V0,3,5:	Leg LM	Arm LM	App LM	Leg LMI	Arm LMI	App LMI	Leg LM/BMI	Arm LM/BMI	App LM/BMI
*N* Included	1254	1254	1254	1242	1242	1242	1238	1238	1238
χ^2^	15.82	6.45	24.90	15.17	6.57	21.04	6.75	3.53	10.09
df	5	5	5	5	5	5	5	5	5
χ^2^ *p* Value	0.007	**0.265**	<0.001	0.010	**0.255**	0.001	**0.240**	**0.620**	**0.073**
SRMR	**0.006**	**0.005**	**0.006**	**0.005**	**0.005**	**0.005**	**0.006**	**0.006**	**0.006**
RMSEA	**0.042**	**0.015**	**0.056**	**0.040**	**0.016**	**0.051**	**0.017**	**<0.001**	**0.029**
RMSEA Lower	**0.020**	**<0.001**	**0.036**	**0.018**	**<0.001**	**0.030**	**<0.001**	**<0.001**	**<0.001**
RMSEA Upper	**0.065**	**0.044**	**0.079**	**0.065**	**0.045**	**0.074**	**0.046**	**0.033**	**0.054**
BB‐NFI	**0.999**	**1.000**	**0.998**	**0.999**	**0.999**	**0.998**	**0.999**	**1.000**	**0.999**
Convergence	Yes	Yes	Yes	Yes	Yes	Yes	Yes	Yes	Yes
Errors	None	None	None	None	None	None	None	None	None

Hu and Bentler's^28^ guidelines for path analysis fit statistics were used to evaluate adequacy of fit.^(^ ^28^ ^)^ Bold indicates significant model fit parameters. No convergence errors or nonpositive definite matrices were observed.

APP = appendicular; BB‐NFI = Bentler–Bonett normed fit index; df = degree of freedom; LM = lean mass; LMI = lean mass index; RMSEA = root mean square error of approximation; SRMR = standardized root mean residual; V0 = baseline; V3 = 48 months; V5 = 120 months.

**Table 4:** Standardized Path Coefficients for Bone and Muscle Predictors of Subsequent Outcomes for Lean Mass and Lean Mass Index at the Leg

LEG LM				
Predictor	Outcome	Estimate	SE	*p* Value
V0 Leg LM	V0 Leg BMD	**0.3339**	**0.0482**	**<0.001**
V0 Leg LM	V3 Leg BMD	**0.0988**	**0.0363**	**0.007**
V0 Leg BMD	V3 Leg LM	0.0022	0.0091	0.805
V3 Leg LM	V3 Leg BMD	**−0.1152**	**0.0339**	**0.001**
V3 Leg LM	V5 Leg BMD	0.0028	0.0353	0.937
V3 Leg BMD	V5 Leg LM	0.0013	0.0113	0.908
V5 Leg LM	V5 Leg BMD	**0.0767**	**0.0321**	**0.017**
V0 Leg BMD	V3 Leg BMD	**1.0309**	**0.0200**	**<0.001**
V3 Leg BMD	V5 Leg BMD	**0.9040**	**0.0089**	**<0.001**
V0 Leg LM	V3 Leg LM	**0.9171**	**0.0239**	**<0.001**
V3 Leg LM	V5 Leg LM	**0.9487**	**0.0202**	**<0.001**

LEG LMI				
Predictor	Outcome	Estimate	SE	*p* Value
V0 Leg LMI	V0 Leg BMD	**0.2809**	**0.0409**	**<0.001**
V0 Leg LMI	V3 Leg BMD	**0.0642**	**0.0282**	**0.023**
V0 Leg BMD	V3 Leg LMI	0.0002	0.0121	0.988
V3 Leg LMI	V3 Leg BMD	**−0.0750**	**0.0256**	**0.003**
V3 Leg LMI	V5 Leg BMD	0.0172	0.0277	0.535
V3 Leg BMD	V5 Leg LMI	0.0047	0.0143	0.741
V5 Leg LMI	V5 Leg BMD	**0.0535**	**0.0259**	**0.039**
V0 Leg BMD	V3 Leg BMD	**1.0312**	**0.0201**	**<0.001**
V3 Leg BMD	V5 Leg BMD	**0.9041**	**0.0089**	**<0.001**
V0 Leg LMI	V3 Leg LMI	**0.9106**	**0.0272**	**<0.001**
V3 Leg LMI	V5 Leg LMI	**0.9560**	**0.0207**	**<0.001**

LEG LM/BMI				
Predictor	Outcome	Estimate	SE	*p* Value
V0 Leg LM/BMI	V0 Leg BMD	**0.3044**	**0.0446**	**<0.001**
V0 Leg LM/BMI	V3 Leg BMD	−0.0040	0.0241	0.868
V0 Leg BMD	V3 Leg LM/BMI	−0.0067	0.0148	0.648
V3 Leg LM/BMI	V3 Leg BMD	−0.0104	0.0209	0.619
V3 Leg LM/BMI	V5 Leg BMD	−0.0492	0.0264	0.063
V3 Leg BMD	V5 Leg LM/BMI	0.0087	0.0150	0.559
V5 Leg LM/BMI	V5 Leg BMD	**0.0564**	**0.0257**	**0.028**
V0 Leg BMD	V3 Leg BMD	**1.0440**	**0.0204**	**<0.001**
V3 Leg BMD	V5 Leg BMD	**0.9134**	**0.0087**	**<0.001**
V0 Leg LM/BMI	V3 Leg LM/BMI	**0.9657**	**0.0395**	**<0.001**
V3 Leg LM/BMI	V5 Leg LM/BMI	**0.9822**	**0.0323**	**<0.001**

Parameter estimates were reported across the three waves along with standard error (SE). Reciprocal relationships were explored only through cross lags. Bold = p value ≤ 0.05. LM = lean mass; LMI = lean mass index; V0 = baseline visit; V3 = 48‐month visit; V5 = 120‐month visit.

**Table 5:** Standardized Path Coefficients for Bone and Muscle Predictors of Subsequent Outcomes for Lean Mass and Lean Mass Index at the Arm

ARM LM				
Predictor	Outcome	Estimate	SE	*p* Value
V0 Arm LM	V0 Arm BMD	**0.3401**	**0.0378**	**<0.001**
V0 Arm LM	V3 Arm BMD	**−0.0633**	**0.0295**	**0.032**
V0 Arm BMD	V3 Arm LM	0.0133	0.0124	0.283
V3 Arm LM	V3 Arm BMD	0.0487	0.0272	0.074
V3 Arm LM	V5 Arm BMD	**−0.0559**	**0.0246**	**0.023**
V3 Arm BMD	V5 Arm LM	**0.0444**	**0.0136**	**0.001**
V5 Arm LM	V5 Arm BMD	**0.1056**	**0.0251**	**<0.001**
V0 Arm BMD	V3 Arm BMD	**1.0368**	**0.0225**	**<0.001**
V3 Arm BMD	V5 Arm BMD	**0.9474**	**0.0101**	**<0.001**
V0 Arm LM	V3 Arm LM	**0.7295**	**0.0227**	**<0.001**
V3 Arm LM	V5 Arm LM	**0.7801**	**0.0199**	**<0.001**

ARM LMI				
Predictor	Outcome	Estimate	SE	*p* Value
V0 Arm LMI	V0 Arm BMD	**0.3111**	**0.0346**	**<0.001**
V0 Arm LMI	V3 Arm BMD	**−0.0646**	**0.0263**	**0.014**
V0 Arm BMD	V3 Arm LMI	0.0126	0.0141	0.371
V3 Arm LMI	V3 Arm BMD	**0.0503**	**0.0241**	**0.037**
V3 Arm LMI	V5 Arm BMD	−0.0382	0.0220	0.083
V3 Arm BMD	V5 Arm LMI	**0.0500**	**0.0156**	**0.001**
V5 Arm LMI	V5 Arm BMD	**0.0761**	**0.0221**	**0.001**
V0 Arm BMD	V3 Arm BMD	**1.0368**	**0.0227**	**<0.001**
V3 Arm BMD	V5 Arm BMD	**0.9494**	**0.0102**	**<0.001**
V0 Arm LMI	V3 Arm LMI	**0.7318**	**0.0242**	**<0.001**
V3 Arm LMI	V5 Arm LMI	**0.8121**	**0.0203**	**<0.001**

ARM LM/BMI				
Predictor	Outcome	Estimate	SE	*p*‐value
V0 Arm LM/BMI	V0 Arm BMD	**0.3071**	**0.0323**	**<0.001**
V0 Arm LM/BMI	V3 Arm BMD	−0.0396	0.0222	0.075
V0 Arm BMD	V3 Arm LM/BMI	−0.0197	0.0169	0.245
V3 Arm LM/BMI	V3 Arm BMD	0.0255	0.0204	0.210
V3 Arm LM/BMI	V5 Arm BMD	−0.0256	0.0198	0.195
V3 Arm BMD	V5 Arm LM/BMI	**0.0477**	**0.0165**	**0.004**
V5 Arm LM/BMI	V5 Arm BMD	0.0301	0.0211	0.155
V0 Arm BMD	V3 Arm BMD	**1.0438**	**0.0228**	**<0.001**
V3 Arm BMD	V5 Arm BMD	**0.9561**	**0.0099**	**<0.001**
V0 Arm LM/BMI	V3 Arm LM/BMI	**0.6877**	**0.0265**	**<0.001**
V3 Arm LM/BMI	V5 Arm LM/BMI	**0.7717**	**0.0223**	**<0.001**

Parameter estimates were reported across the three waves along with standard error (SE). Reciprocal relationships were explored only through cross lags. Bold = p value ≤ 0.05. LM = lean mass; LMI = lean mass index; V0 = baseline visit; V3 = 48‐month visit; V5 = 120‐month visit.

**Table 6:** Standardized Path Coefficients for Bone and Muscle Predictors of Subsequent Outcomes for Appendicular (Both Arms and Legs) Lean Mass and Lean Mass Index

ALM				
Predictor	Outcome	Estimate	SE	*p* Value
V0 App LM	V0 App BMD	**0.4175**	**0.0512**	**<0.001**
V0 App LM	V3 App BMD	−0.0342	0.0287	0.234
V0 App BMD	V3 App LM	0.0025	0.0091	0.782
V3 App LM	V3 App BMD	0.0089	0.0264	0.737
V3 App LM	V5 App BMD	−0.0276	0.0269	0.306
V3 App BMD	V5 App LM	0.0065	0.0109	0.550
V5 App LM	V5 App BMD	**0.0948**	**0.0246**	**<0.001**
V0 App BMD	V3 App BMD	**0.9887**	**0.0135**	**<0.001**
V3 App BMD	V5 App BMD	**0.9401**	**0.0062**	**<0.001**
V0 App LM	V3 App LM	**0.9111**	**0.0243**	**<0.001**
V3 App LM	V5 App LM	**0.9326**	**0.0213**	**<0.001**

ALMI				
Predictor	Outcome	Estimate	SE	*p* Value
V0 App LMI	V0 App BMD	**0.3560**	**0.0438**	**<0.001**
V0 App LMI	V3 App BMD	−0.0377	0.0225	0.093
V0 App BMD	V3 App LMI	−0.0010	0.0121	0.934
V3 App LMI	V3 App BMD	0.0196	0.0199	0.324
V3 App LMI	V5 App BMD	−0.0106	0.0209	0.613
V3 App BMD	V5 App LMI	0.0110	0.0140	0.429
V5 App LMI	V5 App BMD	**0.0648**	**0.0196**	**0.001**
V0 App BMD	V3 App BMD	**0.9893**	**0.0136**	**<0.001**
V3 App BMD	V5 App BMD	**0.9410**	**0.0062**	**<0.001**
V0 App LMI	V3 App LMI	**0.9090**	**0.0279**	**<0.001**
V3 App LMI	V5 App LMI	**0.9496**	**0.0226**	**<0.001**

App LM/BMI				
Predictor	Outcome	Estimate	SE	*p* Value
V0 App LM/BMI	V0 App BMD	**0.3764**	**0.0446**	**<0.001**
V0 App LM/BMI	V3 App BMD	**−0.0452**	**0.0178**	**0.011**
V0 App BMD	V3 App LM/BMI	−0.0152	0.0155	0.327
V3 App LM/BMI	V3 App BMD	0.0226	0.0155	0.145
V3 App LM/BMI	V5 App BMD	−0.0304	0.0190	0.109
V3 App BMD	V5 App LM/BMI	0.0208	0.0150	0.166
V5 App LM/BMI	V5 App BMD	**0.0437**	**0.0191**	**0.022**
V0 App BMD	V3 App BMD	**0.9947**	**0.0137**	**<0.001**
V3 App BMD	V5 App BMD	**0.9462**	**0.0060**	**<0.001**
V0 App LM/BMI	V3 App LM/BMI	**0.9458**	**0.0409**	**<0.001**
V3 App LM/BMI	V5 App LM/BMI	**0.9573**	**0.0342**	**<0.001**

Parameter estimates were reported across the three waves along with standard error (SE). Reciprocal relationships were explored only through cross lags. Bold = p value ≤ 0.05. ALM = appendicular lean mass; ALMI = appendicular lean mass index; App = appendicular; LM = lean mass; LMI = lean mass index; V0 = baseline visit; V3 = 48‐month visit; V5 = 120‐month visit.

**Fig 1:**
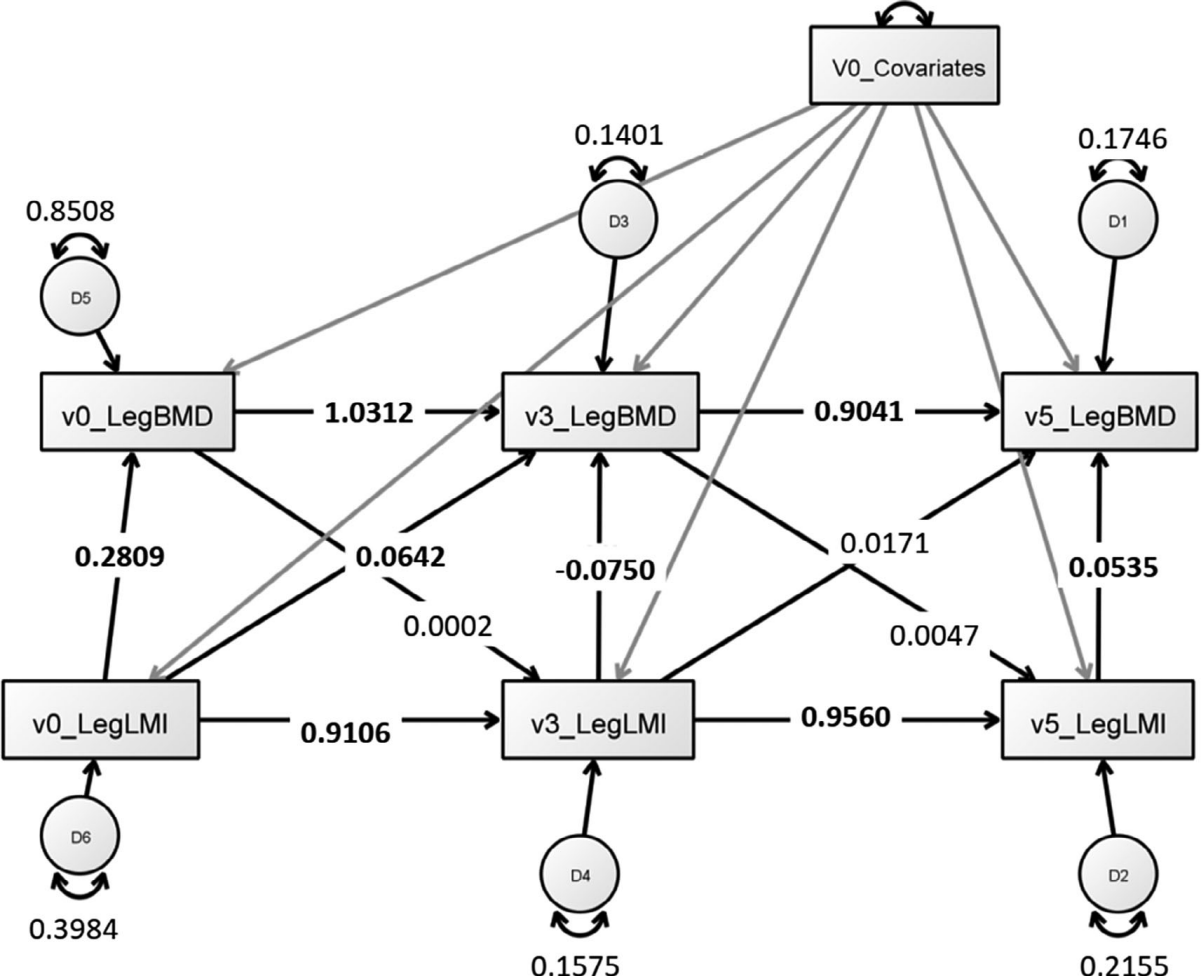
Cross‐lagged panel models of bone–muscle reciprocal relationships over three waves. Contemporaneous muscle–bone effects are larger than any subsequent cross‐lagged effects. Bone lacks any effects on muscle over crossed lags. V0_Covariates shorthand represents directly observed covariates with individual paths to each bone and muscle measure across all time points. LMI = Lean mass index.

The largest muscle–bone relationship was observed for baseline measurements of total body and leg and arm LM, followed by LM/BMI, then LMI. Effects across the whole body appeared larger, in general, compared with both leg and arm sites. For leg but not arm analyses, within time‐point muscle–bone relationships were negative at V3, except after adjusting for BMI, which rendered the association nonsignificant (Table 4). Cross‐lagged effects of LM and LMI on BMD were apparent for legs in the positive direction, but these effects were threefold to 4.5‐fold smaller than contemporaneous effects, and were no longer significant after BMI adjustment (LM/BMI). For arm analyses, cross‐time‐point muscle–bone relationships were negative, but the effect sizes were small (Table 5). These negative relationships remained, even after simplifying models to use the most basic covariates (age, height, weight) without any covariate adjustment, or just by using ordinary least squares regression. A full‐path analysis model, illustrating all relationships including covariate specification and error variances, is providrd in Figure 1. Only arm BMD, but not leg or whole body BMD, showed a cross‐lagged effect on subsequent arm LM, LMI, or LM/LMI, but these associations were weak (standardized B = 0.0444–0.0500). This was validated in linear regression models where a significant effect of V0 BMD on V3 LM or LMI was observed (eg, for LM = 0.861; IQR, 0.708–1.014), but after accounting for repeated V3 BMD in the same model, the relationship was no longer significant (eg, for LM = 0.210; [QR,−0.205 to 0.625), suggesting that any apparent effect of V0 BMD on V3 LM or LMI is only mediated through serial V3 BMD measurement.

Because cross‐lagged relationships (ie, V0 ➔ V3) have the potential to be mediated indirectly through same time‐point relations (ie, V0 LM ➔ V0 BMD) and within‐variable repeated measurements (ie, V0 BMD ➔ V3 BMD), the direct versus indirect mediated effects were compared with one another and expressed as a percentage of the total effect (sum of indirect and direct effects) and as a ratio of one another. In general, indirect effects were larger than the direct effects by 2‐ to 10‐fold (Table 7). By virtue of the presence of some negative direct effects, the indirect effects represented in some cases over 100% of the total effects (Table 8).

**Table 7:** Comparison of Standardized Direct, Indirect, and Total Effects

Predictor	Outcome	Direct	SE	*p* Value	Indirect	SE	*p* Value	Total	SE	*p* Value	Dir % of total	Ind % of total	Ind/dir ratio
V0 Leg LMI	V3 Leg BMD	**0.0642**	**0.0282**	**0.023**	**0.2214**	**0.0485**	**<0.001**	**0.2856**	**0.0405**	**<0.001**	**22.5**	**77.5**	**3.4**
V3 Leg LMI	V5 Leg BMD	0.0172	0.0277	0.535	−0.0167	0.0343	0.626	0.0004	0.0284	0.987	Div by 0	Div by 0	1.0
V0 Leg LM/BMI	V3 Leg BMD	−0.0040	0.0241	0.868	**0.3078**	**0.0512**	**<0.001**	**0.3038**	**0.0446**	**<0.001**	**−1.3**	**101.3**	**DIV by 0**
V3 Leg LM/BMI	V5 Leg BMD	−0.0492	0.0264	0.063	0.0459	0.0321	0.153	−0.0033	0.0266	0.900	Div by 0	Div by 0	0.9
V0 Arm LMI	V3 Arm BMD	**−0.0646**	**0.0263**	**0.014**	**0.3596**	**0.0408**	**<0.001**	**0.2950**	**0.0342**	**<0.001**	**−21.9**	**121.9**	**5.6**
V3 Arm LMI	V5 Arm BMD	−0.0382	0.0220	0.083	**0.1098**	**0.0303**	**<0.001**	**0.0716**	**0.0244**	**0.003**	**−53.4**	**153.4**	**2.9**
V0 Arm LM/BMI	V3 Arm BMD	−0.0396	0.0222	0.074	**0.3379**	**0.0372**	**<0.001**	**0.2983**	**0.0318**	**<0.001**	**−13.3**	**113.3**	**8.5**
V3 Arm LM/BMI	V5 Arm BMD	−0.0256	0.0198	0.195	0.0476	0.0264	0.0711	0.0220	0.0212	0.299	−116.4	216.4	1.9
V0 ALMI	V3 App BMD	−0.0377	0.0224	0.093	**0.3700**	**0.0473**	**<0.001**	**0.3323**	**0.0437**	**<0.001**	−11.3	111.3	9.8
V3 ALMI	V5 App BMD	−0.0106	0.0209	0.613	**0.0800**	**0.0269**	**0.003**	**0.0695**	**0.0226**	**0.002**	−15.3	115.1	7.5
V0 ALM/BMI	V3 App BMD	**−0.0452**	**0.0178**	**0.011**	**0.3956**	**0.0472**	**<0.001**	**0.3504**	**0.0448**	**<0.001**	−12.9	112.9	8.8
V3 ALM/BMI	V5 App BMD	−0.0304	0.0190	0.109	**0.0632**	**0.0238**	**0.008**	**0.0328**	**0.0200**	**0.101**	−92.7	192.7	2.1

Indirect effects account for both parallel mediation paths (ie, V0 muscle ➔ V3 muscle ➔ V3 bone; or V0 muscle ➔ V0 bone ➔ V3 bone ➔ Same for V3 ➔ V5 effects). Pink indicates negative effects. Blue indicates positive effects. Bold indicates significant results. Div by 0 indicates uninterpretable values because of division by 0. An ind/dir ratio >1.0 indicates a larger absolute value of indirect versus a direct effect. ALM = appendicular lean mass; ALMI = appendicular lean mass index; Dir = direct; Div = divide; Ind = Indirect; LM = lean mass; LMI = lean mass index; V0 = baseline visit; V3 = 48‐month visit; V5 = 120‐month visit.

**Table 8:** Unstandardized Path Coefficients for Bone and Muscle Predictors of Subsequent Outcomes for Lean Mass and Lean Mass Index at the Leg

LEG LM				
Predictor	Outcome	Estimate	SE	*p* Value
V0 Leg LM	V0 Leg BMD	**0.0316**	**0.0046**	**<0.001**
V0 Leg LM	V3 Leg BMD	**0.0093**	**0.0034**	**0.007**
V0 Leg BMD	V3 Leg LM	0.0238	0.0966	0.805
V3 Leg LM	V3 Leg BMD	**−0.0108**	**0.0032**	**0.001**
V3 Leg LM	V5 Leg BMD	0.0003	0.0038	0.937
V3 Leg BMD	V5 Leg LM	0.0136	0.1172	0.908
V5 Leg LM	V5 Leg BMD	**0.0085**	**0.0035**	**0.017**
V3 Leg BMD	V5 Leg BMD	**1.0302**	**0.0159**	**<0.001**
V3 Leg LM	V5 Leg LM	**0.9226**	**0.0219**	**<0.001**

LEG LMI				
Predictor	Outcome	Estimate	SE	*P* Value
V0 Leg LMI	V0 Leg BMD	**0.0968**	**0.0143**	**<0.001**
V0 Leg LMI	V3 Leg BMD	**0.0221**	**0.0097**	**0.023**
V0 Leg BMD	V3 Leg LMI	0.0005	0.0355	0.988
V3 Leg LMI	V3 Leg BMD	**−0.0255**	**0.0087**	**0.003**
V3 Leg LMI	V5 Leg BMD	0.0066	0.0107	0.535
V3 Leg BMD	V5 Leg LMI	0.0134	0.0407	0.741
V5 Leg LMI	V5 Leg BMD	**0.0214**	**0.0104**	**0.039**
V3 Leg BMD	V5 Leg BMD	**1.0297**	**0.0159**	**<0.001**
V3 Leg LMI	V5 Leg LMI	**0.9231**	**0.0236**	**<0.001**

LEG LM/BMI				
Predictor	Outcome	Estimate	SE	*p* Value
V0 Leg LM/BMI	V0 Leg BMD	**0.8942**	**0.1329**	**<0.001**
V0 Leg LM/BMI	V3 Leg BMD	−0.0117	0.0705	0.868
V0 Leg BMD	V3 Leg LM/BMI	−0.0023	0.0050	0.648
V3 Leg LM/BMI	V3 Leg BMD	−0.0308	0.0620	0.619
V3 Leg LM/BMI	V5 Leg BMD	−0.1665	0.0893	0.062
V3 Leg BMD	V5 Leg LM/BMI	0.0029	0.0049	0.559
V5 Leg LM/BMI	V5 Leg BMD	**0.1965**	**0.0895**	**0.028**
V3 Leg BMD	V5 Leg BMD	**1.0412**	**0.0160**	**<0.001**
V3 Leg LM/BMI	V5 Leg LM/BMI	**0.9533**	**0.0342**	**<0.001**

Parameter estimates were reported across the three waves along with standard error (SE). Reciprocal relationships were explored only through cross lags. Bold indicates significant results. Bold = p value ≤ 0.05. LM = lean mass; LMI = lean mass index; V0 = baseline visit; V3 = 48‐month visit; V5 = 120‐month visit.

In post hoc analyses, baseline leg LM and LMI, but not arm LM and LMI (LM/BMI was not significant), were marginally associated with subsequent changes in leg BMD from 53 to 127 months (LM = 0.0044; IQR, −0.0002 to 0.0090; *p* = 0.060; LMI = 0.0134; IQR, −0.0006 to 0.0274; *p* = 0.061), but both of these relationships were ablated after further accounting for baseline leg BMD, which was in fact associated with subsequent changes in leg BMD (0.035; IQR, 0.007–0.064). In contrast, lower baseline arm BMD, but not leg BMD, was significantly associated with larger declines in LM, LMI, and LM/BMI from 53 to 127 months (0.0355 [IQR, 0.0095–0.0615] kg lower LM per 0.1 g/cm^2^ lower BMD; 0.0113 [IQR, 0.0024–0.0202] kg/m^2^ lower LMI per 0.1 g/cm^2^ lower BMD; and 0.1329 [IQR, 0.0313–0.2345] kg per kg/m^2^ lower LM/BMI per 0.1 g/cm^2^ lower BMD) after accounting for baseline arm LM or LMI. Unlike the positive associations observed between baseline arm BMD and subsequent changes in arm BMD from V3 to V5, larger arm LM, LMI, and LM/BMI values were associated with declines in respective LM (−0.0213 [IQR, −0.0251 to −0.0174]), LMI (−0.0207 [IQR, −0.0247 to −0.0167]), and LM/BMI (−0.2311 [IQR, −0.2750 to −0.1871]) measures from V3 to V5 (Table 9).

**Table 9:** Unstandardized Path Coefficients for Bone and Muscle Predictors of Subsequent Outcomes for Lean Mass and Lean Mass Index at the Arm

ARM LM				
Predictor	Outcome	Estimate	SE	*p* Value
V0 Arm LM	V0 Arm BMD	**0.0371**	**0.0042**	**<0.001**
V0 Arm LM	V3 Arm BMD	**−0.0068**	**0.0032**	**0.031**
V0 Arm BMD	V3 Arm LM	0.1263	0.1176	0.283
V3 Arm LM	V3 Arm BMD	0.0051	0.0028	0.073
V3 Arm LM	V5 Arm BMD	**−0.0063**	**0.0028**	**0.023**
V3 Arm BMD	V5 Arm LM	**0.4143**	**0.1263**	**0.001**
V5 Arm LM	V5 Arm BMD	**0.0123**	**0.0029**	**<0.001**
V3 Arm BMD	V5 Arm BMD	**1.0251**	**0.0158**	**<0.001**
V3 Arm LM	V5 Arm LM	**0.7567**	**0.0211**	**<0.001**

ARM LMI				
Predictor	Outcome	Estimate	SE	*p* Value
V0 Arm LMI	V0 Arm BMD	**0.1138**	**0.0129**	**<0.001**
V0 Arm LMI	V3 Arm BMD	**−0.0233**	**0.0095**	**0.014**
V0 Arm BMD	V3 Arm LMI	0.0360	0.0403	0.371
V3 Arm LMI	V3 Arm BMD	**0.0175**	**0.0083**	**0.036**
V3 Arm LMI	V5 Arm BMD	−0.0143	0.0082	0.083
V3 Arm BMD	V5 Arm LMI	**0.1353**	**0.0422**	**0.001**
V5 Arm LMI	V5 Arm BMD	**0.0303**	**0.0088**	**0.001**
V3 Arm BMD	V5 Arm BMD	**1.0241**	**0.0159**	**<0.001**
V3 Arm LMI	V5 Arm LMI	**0.7622**	**0.0217**	**<0.001**

ARM LM/BMI				
Predictor	Outcome	Estimate	SE	*p* Value
V0 Arm LM/BMI	V0 Arm BMD	**1.1093**	**0.1190**	**<0.001**
V0 Arm LM/BMI	V3 Arm BMD	−0.1414	0.0790	0.074
V0 Arm BMD	V3 Arm LM/BMI	−0.0057	0.0049	0.245
V3 Arm LM/BMI	V3 Arm BMD	0.0871	0.0694	0.210
V3 Arm LM/BMI	V5 Arm BMD	−0.0943	0.0727	0.195
V3 Arm BMD	V5 Arm LM/BMI	**0.0130**	**0.0045**	**0.004**
V5 Arm LM/BMI	V5 Arm BMD	0.1185	0.0833	0.155
V3 Arm BMD	V5 Arm BMD	**1.0310**	**0.0159**	**<0.001**
V3 Arm LM/BMI	V5 Arm LM/BMI	**0.7198**	**0.0235**	**<0.001**

Parameter estimates were reported across the three waves along with standard error (SE). Reciprocal relationships were explored only through cross lags. Bold indicates significant results. Bold = p value ≤ 0.05. LM = lean mass; LMI = lean mass index; V0 = baseline visit; V3 = 48‐month visit; V5 = 120‐month visit.

## Discussion

Among Afro‐Caribbean men over 40 years of age, lean mass of the arms and legs had modest short‐term effects on BMD within the same region of interest. Although cross‐lagged effects were 3.0‐ to 4.5‐fold smaller, they lasted up to 53 months, but dissipated after 74 months. Most lagged effects were mediated primarily through same time‐point lean mass–bone relations and subsequent repeated measure correlations, rather than a true direct lagged effect. Short‐term associations appeared the largest for whole‐body measures. Short‐term effects, but not long‐term effects, were therefore most prominently observed within lean and bone mass data obtained from DXA. There was only a weak reciprocal effect of BMD on future lean mass measures with or without adjustment for height or BMI, but this was found only at the arm. Within the same time period, effects of bone mass on lean mass were not distinguishable from the effects of lean mass on bone mass (Table 10).

**Table 10:** Unstandardized Path Coefficients for Bone and Muscle Predictors of Subsequent Outcomes for Appendicular (Both Arms and Legs) Lean Mass and Lean Mass Index

ALM				
Predictor	Outcome	Estimate	SE	*p* Value
V0 App LM	V0 App BMD	**0.0106**	**0.0013**	**<0.001**
V0 App LM	V3 App BMD	−0.0009	0.0008	0.233
V0 App BMD	V3 App LM	0.1009	0.3641	0.782
V3 App LM	V3 App BMD	0.0002	0.0007	0.737
V3 App LM	V5 App BMD	−0.0008	0.0007	0.306
V3 App BMD	V5 App LM	0.2532	0.4233	0.550
V5 App LM	V5 App BMD	**0.0026**	**0.0007**	**<0.001**
V3 App BMD	V5 App BMD	**1.0183**	**0.0107**	**<0.001**
V3 App LM	V5 App LM	**0.9302**	**0.0230**	**<0.001**

ALMI				
Predictor	Outcome	Estimate	SE	*p* Value
V0 App LMI	V0 App BMD	**0.0328**	**0.0041**	**<0.001**
V0 App LMI	V3 App BMD	−0.0036	0.0021	0.092
V0 App BMD	V3 App LMI	−0.0111	0.1348	0.934
V3 App LMI	V3 App BMD	0.0018	0.0018	0.324
V3 App LMI	V5 App BMD	−0.0011	0.0021	0.613
V3 App BMD	V5 App LMI	0.1180	0.1491	0.429
V5 App LMI	V5 App BMD	**0.0066**	**0.0020**	**0.001**
V3 App BMD	V5 App BMD	**1.0175**	**0.0108**	**<0.001**
V3 App LMI	V5 App LMI	**0.9345**	**0.0251**	**<0.001**

App LM/BMI				
Predictor	Outcome	Estimate	SE	*p* Value
V0 App LM/BMI	V0 App BMD	**0.3105**	**0.0375**	**<0.001**
V0 App LM/BMI	V3 App BMD	**−0.0383**	**0.0150**	**0.011**
V0 App BMD	V3 App LM/BMI	−0.0185	0.0189	0.327
V3 App LM/BMI	V3 App BMD	0.0190	0.0131	0.145
V3 App LM/BMI	V5 App BMD	−0.0277	0.0173	0.109
V3 App BMD	V5 App LM/BMI	0.0245	0.0177	0.166
V5 App LM/BMI	V5 App BMD	**0.0401**	**0.0175**	**0.022**
V3 App BMD	V5 App BMD	**1.0232**	**0.0108**	**<0.001**
V3 App LM/BMI	V5 App LM/BMI	**0.9510**	**0.0365**	**<0.001**

Parameter estimates were reported across the three waves along with standard error (SE). Reciprocal relationships were explored only through cross lags. Bold indicates significant results. Bold = p value ≤ 0.05. App = appendicular; LM = lean mass; LMI = lean mass index; V0 = baseline visit; V3 = 48‐month visit; V5 = 120‐month visit.

### Timing and effect size of muscle–bone relationships

The significant effect of LM, LMI, and LM/BMI on BMD at various sites supported our hypothesis of muscle's short‐term effects on bone. The fact that we did not observe any long‐lasting effects of muscle on bone beyond 53 months suggests that muscle–bone interactions may be regulated over shorter periods. Gaining muscle mass may only be beneficial for the short term, and any muscle losses may also be reflected in bone more immediately. In middle‐aged men and women, Culvenor and colleagues saw a correlation between 2‐year changes in muscle and bone (*r* = 0.75 for men, *r* = 0.68 for women).^(^ ^30^ ^)^ Similarly, Ruff and colleagues showed correlations in growth velocity in femoral strength with thigh muscle size (*R* ^2^ = 0.10–0.25), but in a limited sample of 20 younger adults.^(^ ^31^ ^)^ In a 2‐year study of 248 healthy girls applying multivariable regression models, Farr and colleagues saw that higher 2‐year changes in muscle density were associated with greater 2‐year gains in cortical (*r* = 0.10) and trabecular (*r* = 0.25) volumetric BMD of the tibia using pQCT.^(^ ^32^ ^)^ However, each of these studies measured parallel changes and did not quantify the directionality of the bone–muscle relationships, and did not adjust for contemporaneous associations, which could yield very different results. Indeed, we showed that in general linear models, accounting for baseline versions of the outcomes for which change was predicted resulted in an ablation or abolishment of these effects. One Korean study,^(8)^ which reported the ability of leg lean mass to predict changes in total hip BMD over 5 years, evaluated this relationship using lean mass predictors measured at the same time as the initial point of BMD change. This model likely induced an artificial effect based on the correlation between lean mass and total hip BMD measured at the same time point. For this reason, we examined cross‐lagged effects, while also considering contemporaneous associations. To demonstrate this point, we repeated the analyses this group had done (effect of V0 LM on change in BMD from V3–V5) and did not observe a relationship when using baseline LM predictors (0.0033 [IQR, −0.0013 to 0.0080], *p* = 0.160), but induced a relationship (0.0083 [IQR, 0.0039–0.0127], *p* < 0.001) when using predictors coinciding with the same time point as the start time of the change (effect of V3 LM on change in BMD from V3–V5).

Although the effect size for muscle's impact on bone appears modest, whether contemporaneous or cross‐lagged, a similar magnitude of effect was also previously observed in DXA body‐composition analyses conducted by other groups. In over 400 middle‐aged Indian women, Marwaha and colleagues found similar effects of leg lean mass and femoral BMD of 0.066 (*p* = 0.032).^(^ ^33^ ^)^ Verschueren and colleagues also saw a comparably small effect of appendicular lean mass on total hip BMD (0.064, *p* < 0.001) among men 40 to 79 years old in the United Kingdom.^(^ ^34^ ^)^ Chalhoub and colleagues studied men 65 years and older, finding only small differences in midtibia cortical thickness and femoral neck BMD across quartiles of appendicular lean mass, representing <5% of the mean value of bone measures.^(^ ^35^ ^)^ Reider and colleagues also saw modest cross‐sectional relationships between percentage lean mass and femoral stress as measured using hip structural analysis of leg DXA images (*r* = −0.123 [IQR, −0.139 to –0.106]), representing 1.6% of mean femoral stress (6.29 MPa).^(^ ^36^ ^)^ Meanwhile, our largest effects observed represented up to 6.9% of the mean BMD of the leg and up to 4.3% of BMD of the arm. Ho‐Pham and colleagues simulated the distribution of lean mass effects on lumbar spine BMD and showed that these small effects (centered around 0.03 g/cm^2^ per kg of lean mass) remained within this neighborhood of magnitude across a range of sample sizes with only larger differences in CIs.^(^ ^37^ ^)^ The fact that muscle–bone coefficients were larger across the whole body than at either the legs or arms suggests there may be non‐overlapping effects that contribute to larger overall associations across all appendicular sites.

### Lack of reciprocal bone effect on muscle

Although there has been molecular evidence of osteocyte secreted factors having an effect on myoblasts,^(^ ^12^ ^)^ bone's impact on muscle as represented here as BMD and LM was weak and only apparent within the arm‐related CLPMs of our long‐term DXA cohort study. The lack of effect observed over 54 and 73 months for other sites could be explained by the long durations of follow‐up and the possibility that these effects are more short term. However, we were unable to tease‐out bone's impact on muscle from the opposite direction of causality within contemporaneous associations because of the mathematical equivalency of these simultaneous relationships. Yoshimura and colleagues in fact saw this reciprocal effect of bone on muscle over 4 years (OR, 2.99; 95% CI, 1.46–6.12; *p* < 0.01)—a shorter period than examined here—but no effect of muscle on bone during the same period (OR, 2.11; 95% CI, 0.59–7.59; *p* = 0.25). However, rather than examining site‐specific associations, general osteoporosis (WHO definition at hip and spine) and sarcopenia (Asian Working Group) definitions were used, combining a variety of muscle metrics derived at both upper and lower extremities.^(^ ^38^ ^)^ Although we saw a heterogeneity of effects between the arms and legs in earlier muscle–bone analyses, it was not surprising to also see here bone's effect on muscle changes only at the arm and not at the legs. Perhaps weight‐bearing may be a potential moderator of muscle's effects on bones versus the opposite. We suggest future avenues of research to explore the role of weight‐bearing on bone–muscle reciprocal relations using shorter‐term longitudinal cohort data.

### Declines in bone and muscle properties over time

Based on reference curve leg LMI values from NHANES (the National Health and Nutrition Examination Survey; 1999–2004)^(^ ^39^ ^)^ for black men 40 years of age (L = 0.118, M = 3.457, S = 0.147), the baseline *z*‐score comparison with the current cohort was −0.05, indicating that the cohort was well within norms. By 127 months postbaseline, the mean *z*‐score had changed to −0.37, which remained within acceptable normative limits. Similarly, for arm LMI (L = 0.756, M = 1.425, S = 0.156), the baseline *z*‐score was −0.02 and progressed to −0.39 after 127 months. Although lean mass appeared to decline at similar rates for arms and legs, the arms showed twice faster decline compared to legs based on annual percentage changes. Kim and colleagues also reported annual percentage changes averaged over 5 years for Korean men of −0.58%,^(^ ^8^ ^)^ similar to the −0.76% reported in the Afro‐Caribbean men in this Tobago cohort. The more rapid decline between V3 and V5 versus V0 and V3, after accounting for time elapsed, may suggest that the rates of changes in bones and muscles may be nonlinear, although there are insufficient data points available to ascertain this postulation. Future studies should quantify serial changes in bone and muscle properties to better examine parallel trajectories in both tissues.

### Advantage of cross‐lagged panel models

The CLPMs constructed here have the benefit of not only specifying cross‐lagged relations, but concurrently accounting for contemporaneous associations and covariance between time points that are unexplained by specified paths. Linear regression models fail to properly account for repeated measures and same time point effects, thus missing potential parallel mediation paths and failing to isolate direct from indirect effects. One of the disadvantages, however, is the occasional lack of convergence and the need to use alternative model optimization techniques, which did occur for models examining all appendicular sites together. Although the fact that nonconvergence occurred may be a sign that arm and leg associations needed to be treated separately, which in fact was well‐supported by the differing associations observed here. Although negative errors and unexpected directionality of paths could be a symptom of model misspecification, we replicated the panel models with simpler specifications, including using fewer or no covariates, and still observed some unexpected negative effects (V0 arm muscle on V3 arm bone). The same negative effects were also observed in replicated linear regression models, which suggests it is not a symptom of poor model specification, but may reflect some reality regarding the data. Alternatively, it is possible there is some feedback mechanism that may work through other pathways that have not been fully described in these models.

### Study strengths and limitations

This study benefited from a large sample size with long‐term follow‐up and serial DXA measures, as well as a focus on an understudied ethnic group. The analyses also demonstrated robustness in parsimony, absolute, and relative fit, with few missing data for variables. The ability to describe reciprocal effects was a major strength of this study, although the lack of more short‐term bone and muscle repeated measurements within a one‐year time frame prevented our ability to observe potentially stronger muscle–bone relationships. In fact, with longer follow‐up periods, it is difficult to fully account for the contribution of a larger gamut of events such as significant weight loss, physical (in)activity, hospitalization, and dietary changes. In our analyses, we were only able to account for alcohol use, comorbidities, hospitalization, grip strength, and walking speed as an estimate of physical function. The mean age of the cohort is also younger (53 ± 9 years), though sarcopenia and osteopenia tend to be more prominent in older adults. Although DXA is a standard‐of‐care modality and has been widely used to examine total body composition, the lean mass measurements examined here do not directly measure skeletal muscle; the DXA scanners were originally calibrated for optimal computation of BMD rather than lean mass. DXA's ability to measure lean tissue or apparent “muscle” density (similar to BMD, ie, lean mass divided by area) could be useful to reflect fat distribution, but this metric is not a standard output from the manufacturer's software. Instead, height squared is thought to account for surface area of the body to yield an approximation for mass distribution over area, and adjustment for BMI is thought to account for fat distribution. However, we do not know the proportion of the arms and legs as a function of total body surface area (represented by height squared). Therefore, adjustment for the entirety of height squared or BMI is not entirely accurate. This study focused on bone and lean mass and not function, with adjustment only for grip strength and walking speed, but not other functional measures because of their unavailability in the cohort. In fact, serial measures of muscle function and their cross‐lagged effects on bone have not been examined. Lean mass and function are, however, highly correlated, thus making it difficult to account for both in models without encountering a high degree of collinearity in panel models. Though also a strength, these results, which focus on men of Afro‐Caribbean ancestry, may limit applicability to women or those of other ethnic groups; though we saw similar declines in bone–muscle properties in other ethnic groups such as Koreans.^(^ ^8^ ^)^ This study was also potentially limited to examining gross lean mass and bone mass characteristics obtained by DXA through longitudinal data available over longer periods (up to 127 months). Future studies should exercise better technologies, including CT or deuterated water/creatine experiments, to more directly study muscle composition, including muscle function in shorter‐term (several months) bone–muscle relations.

In summary, the benefits of muscle in keeping bone strong may not be long‐lasting without continued stimulation. Efforts to maintain muscle mass and bone mass might be more effective with shorter‐term monitoring. Muscle–bone impacts and BMD declines at the arms being larger than at the legs suggest that stronger interventions should be focused on arm strengthening. Although we did not observe sizeable bone effects on muscle, the ability of arm BMD to predict future changes in arm lean mass suggests that there may be some value in using current BMD, at least at the arms, as a risk factor for sarcopenia. More investigation into muscle‐bone relationships over weeks rather than months or years may reveal stronger associations.

## Disclosures

The authors have no conflicts of interest to declare

## AUTHOR CONTRIBUTIONS

**Andy KO Wong:** Methodology; investigation; analysis: writing original draft; review and editing; submission. **Anthony Pokhoy:** Writing‐original draft; writing‐review and editing. **Abinaa Chandrakumar:** Project administration; writing‐original draft; writing‐review and editing. **Ryan Cvejkus:** Data curation; funding acquisition; investigation; methodology; project administration; resources; writing‐review and editing. **Joseph Zmuda:** Data curation; funding acquisition; investigation; methodology; project administration; resources; writing‐review and editing.

### PEER REVIEW

The peer review history for this article is available at https://publons.com/publon/10.1002/jbm4.10406.
